# Supplementation with 2′-fucosyllactose, a prebiotic human milk oligosaccharide (HMO), in a magnesium-containing medical food reduces chemotherapy-induced mucositis in Wistar rats

**DOI:** 10.3389/fnut.2026.1859479

**Published:** 2026-06-25

**Authors:** Sourabh Kharait, Matthew Wilcox, Kyle Stockdale-Stanforth, Vishnu Thakare

**Affiliations:** 1 IGH Naturals, Inc., Rocklin, CA, United States; 2 Department of Medicine, Columbia University Medical Center, New York, NY, United States; 3 Aelius Biotechnology, Newcastle upon Tyne, United Kingdom; 4 Scitesla Research Private Limited, Navi Mumbai, India

**Keywords:** 2′-fucosyllactose, chemotherapy, human milk oligosaccharides, magnesium, microbiome, mucositis

## Abstract

Mucositis is a common debilitating complication of chemotherapy in cancer patients that limits enteral nutrition, causes diarrhea, dehydration and electrolyte wasting. 2’-fucosyllactose (2’-FL), a non-digestible oligosaccharide initially isolated from human milk, is critical in the development of gastrointestinal function and microbiome maturation in the newborn. Here, we demonstrate, that supplementation of 2’-FL in a medical food with magnesium (Humolyte®), protects Wistar rats from gastrointestinal mucosal injury from chemotherapeutic drugs doxorubicin, irinotecan, 5-flurouracil, and cisplatin. Supplementation with Humolyte® reduced weight loss and the severity of diarrhea from chemotherapy. Histopathology of ileal and colonic tissue showed preservation of overall mucosal anatomy including goblet cells and a markedly lesser inflammation. Humolyte® reduced hypertrophy and edema of oral mucosa caused by chemotherapy. *In vitro*, Humolyte® improved goblet cell survival and mucin secretion while reducing monolayer permeability. Thus, Humolyte® can be a useful adjunctive therapy for patients with cancer suffering from chemotherapy-induced mucositis.

## 1 Introduction

Chemotherapy is often prescribed for patients with solid tumors of the head and neck, lung, breast, and ovary, and also for hematologic malignancies. High-dose chemotherapy is particularly effective in the preparation of patients for a bone marrow transplant (1). However, these agents have significant off-target toxicity on both the bone marrow and the gastrointestinal (GI) tract (2). GI toxicity can affect the mucosal lining from the oral cavity to the anal canal and often manifests as chemotherapy-induced mucositis (CIM), presenting with oral ulcers and/or symptoms such as diarrhea and abdominal pain (3). In majority of patients, intestinal mucositis may lead to significant dehydration and electrolyte imbalance, particularly due to magnesium and potassium (4).

Oral mucositis (stomatitis) is a painful and particularly severe in patients who receive both chemotherapy and radiation, as in the case of head and neck cancers (5). Odynophagia further compounds dehydration and malnutrition due to a poor oral intake (6). These patients often suffer from pre-renal azotemia or acute kidney injury leading to temporary cessation or dose reductions of chemotherapy, which may render the cancer more aggressive (7). These side effects can be a significant deterrence to compliance with chemotherapy. Prior studies have indicated that hypomagnesemia from dehydration in chemotherapy is associated with poor clinical outcomes including worsening of pain (8, 9). Management of hypomagnesemia remains a clinical challenge since most magnesium supplements, particularly magnesium oxide, can severely worsen diarrhea when taken at high doses. An injured GI tract is particularly prone to continuous magnesium wasting due to poor paracellular transport and an impaired absorptive capacity, along with renal wasting through a direct effect on transient receptor potential melastatin 6 (TRPM6) channels (10). While sodium and potassium are relatively easy to replete through diet, magnesium deficiency in cancer patients can worsen quickly and remains a commonly unmet need (11). Consequently, these patients often require intravenous (IV) hydration with saline and electrolytes, increasing healthcare costs without any improvement in clinical outcomes. The frequent need for IV hydration requires invasive procedures such as placement of port-a-caths that increase infection risk. Presently, few therapies that can prevent or reduce the severity of mucositis and its related complications (such as pain and dehydration) (12). Thus, strategies to prevent GI epithelial injury represent a novel approach in reducing chemotherapy-induced dehydration and improving patient outcomes (13, 14).

At a cellular level, mucositis disrupts the GI cell–cell adhesions through a direct fragmentation of the intraepithelial tight junctions (15). In severe cases, this leads to epithelial detachment from the basement membrane, followed by dissemination of inflammatory and infectious particles from the GI lumen into the systemic circulation (16). This triggers an influx of inflammatory cells and the production of chemokines such as interleukins (IL)-1β, IL-6, and tumor necrosis factor-*α* (TNF-α) (17). The resulting damage from inflammation leads to ulceration, before stimulating the process of healing (5, 16). During this cycle, significant loss of water, electrolytes, and nutrients can manifest as malnutrition, leading to debility and pain. Local therapies such as ice chips or saline rinses are inadequate in controlling pain or promoting healing of mucositis (2). Thus, newer therapies that can augment healing of the mucosal injury or reduce the extent of mucosal damage from chemotherapy are desperately needed.

Human milk oligosaccharides (HMOs), first isolated from human milk, are complex non-digestible carbohydrates that enable the maturation of the infant newborn’s gut epithelial lining in first few weeks of life (18). They play a crucial role as food for the commensal bacteria, particularly the bifidobacterial species (19, 20), leading to the development of a healthy gut microbiome during childhood (18, 21). Recent studies have shown that certain HMOs may significantly protect the GI barrier function of animals exposed to toxic metabolites including some chemotherapeutic agents (22) and against certain viral infections in humans (23–26). HMOs such as 2′-fucosyllactose (2′-FL), also help in improving GI barrier function by improving cell–cell adhesion and decreasing intestinal permeability (25), and have been used in infant nutrition to improve gut health (27–29). This study hypothesized that 2′-FL, the most abundant HMO, administered in a magnesium-rich rehydration mix, Humolyte^®^, can protect the GI tract in animals subjected to chemotherapy and reduce the subsequent dehydration and electrolyte deficiencies by minimizing diarrhea and improving bowel function. Furthermore, this study demonstrates that such a protective effect on the oral and GI epithelium is drug-agnostic and is seen against all commonly used chemotherapeutic agents that cause mucositis. This study also elucidate the mechanism by which Humolyte^®^ can improve GI cellular function through stabilization of the barrier function and upregulation of mucin secretion through the presence of the HMO. The medical food containing 2′-FL has significant potential in reducing the off-target GI side effects in chemotherapy and provide an easier way of electrolyte repletion orally while minimizing mucositis symptoms in humans. This study provides preliminary data to inform the design of a clinical trial in patients with cancer.

## 2 Materials and methods

### 2.1 Materials

Chemotherapeutic drugs used in this study were obtained from the following pharmaceuticals: Doxorubicin (Zubidox^®^) from RPG Life Sciences, India; irinotecan (Irinotel^®^) from Fresenius Kabi Oncology Limited, India; 5-flurouracil (Oncofluor^®^500) from United Biotech, India; and Cisplatin (Cistero^®^) from Hetero Healthcare, India. Humolyte^®^ medical food was provided by IGH Naturals, Inc. Rocklin, CA, USA. Table 1 provides details about nutrient concentrations in the Humolyte^®^ medical food. Dosing of Humolyte^®^ was calculated based on the target concentration of the active ingredient 2′-FL present in physiological concentrations in human milk (5–8 mg/mL). To obtain a 6 mg/mL of final concentration of 2′-FL, 13.5 g of Humolyte^®^ was mixed in 1 L of drinking water and supplemented to animals in the treatment group (see Table 1). Animals were randomized to receive water (control) or Humolyte^®^ beginning 5 days prior to the injection of chemotherapeutic drugs on day 6 and continued till day 10. The doses of chemotherapeutic drugs utilized in this experiment were as follows: doxorubicin 10 mg/kg, 5-flurouracil at 400 mg/kg, irinotecan at 200 mg/kg, and cisplatin at a dose of 5 mg/kg. All animals received a single intraperitoneal injection based on their body weight on day 6. These doses have been shown to induce GI mucositis in prior studies (30–33).

**Table 1:** Nutrient and mineral composition of Humolyte^®^ medical food.

Ingredient	Amount per serving (4.5 g) as a powder	Final concentration of nutrients after reconstitution
2′-fucosyllactose	2 g	6 mg/mL
Sodium (as chloride)	200 mg	0.6 mg/mL
Potassium (as citrate)	150 mg	0.45 mg/mL
Magnesium (as citrate)	80 mg	0.24 mg/mL

### 2.2 Animal experiments

Wistar rats were purchased from Lacsmi Biofarm Private Limited, Pune, India and all animals received the recommended institutional care as per the Committee for the Purpose and Supervision of Experiments on Animals (CPCSEA) guidance to care for laboratory animals. The study was approved by the Local Institutional Animal Ethics Committee, (IAEC, Approval number SCI/IAEC/2024-25/92, dated 11 April, 2024). Animal studies were conducted by the clinical research organization, Scitestla Private Limited, Navi Mumbai, India according to all GLP compliant guidelines between 7 May, 2024 through 17 May, 2024 (day 10 of experiment). Animals were housed in standard polycarbonate cages at 23 °C and all care was performed as recommended with drinking water and chow (VRK nutritional solution) ad lib. After 7 days of acclimatization, 8-week old adult Wistar rats were randomized to two treatment groups—the control group (drinking water) and test group (Humolyte^®^, a medical food supplemented with 2′-FL and electrolytes, mixed in drinking water, starting5 days prior to the chemotherapy and continued until euthanasia on day 11 by carbon-dioxide inhalation).

Animals were monitored throughout the experimental period for their dietary intake, activity, and weight. Bowel movement scores were recorded daily based on the assessment of stool consistency and rated on a scale of 0 (well formed) to 4 (profuse watery diarrhea). A score of 1 indicated mild soft but formed stool, 2 indicated mild loose BM, and 3 indicated loose BM. Thus, a higher score denoted severity of the diarrheal state. Animals were euthanised on day 11 using CO_2_ inhalation/overdose technique (30% chamber vol/min). Lack of response to noxious stimuli was first confirmed after CO_2_ inhalation once animals were unresponsive and death was confirmed further using cervical dislocation technique. Animal tissues were subjected to histopathology of the oral mucosa and the gastrointestinal tract, particularly the ileum and colon. After hematoxylin and eosin staining, all slides were read by a pathologist who was blinded to the treatment groups, to assess the extent of tissue damage. A semiquantitative score from 0 (normal architecture) to 4 (severe damage) was assigned by the pathologists for each of the following characteristics for colonic tissue: (a) degree of inflammation characterized by the presence of infiltrative cells (0–4); (b) loss of epithelial cell architecture or integrity measured by necrosis or thrombosis in the mucosa or submucosal tissue, fibrosis, or edema (0–4); and (c) the assessment of mucin secreting apparatus (depth of crypts, the height of the villi, or flattening of brush border); and (d) loss of goblet cells (0–4). The maximal allowable score in each category was 4 and a total composite allowable score was 16. A score of 1 depicted a minimal change, 2 depicted mild, and 3 indicated a moderate change in each category as described above. A higher score directly correlated with the severity of the damage of the epithelial and secretory apparatus and inflammation and was compared between control and Humolyte^®^ treated groups in each of the chemotherapy regimens. After the weight of the entire intestinal tissue (from duodenum to rectum) was recorded for each animal, at least three distinct areas of oral, ileal, and colonic mucosa were sectioned and stained with H/E to capture at least three distinct fields in each sample and were read by the pathologist. Goblet cells were quantified by counting individual cells per high power field (hpf) in at least three distinct areas within a sample; three distinct areas within the colon were sectioned per animal in each group for analysis. Similarly, for the oral mucosa, a score of 0–4 was attributed to each of the pathological features: (a) epithelial erosion or hyperplasia, (b) the extent of inflammation, and (c) vascular congestion and edema with a maximal cumulative score of 12 within each treatment condition.

### 2.3 *In vitro* studies

#### 2.3.1 Cell lines and culture

Caco-2 and HT29-MTX cells were obtained from Sigma Aldrich^®^ and grown to confluence for 21 days as per manufacturer’s instructions in transwell chambers with 200 μL of Dulbecco’s Modified Eagle Medium (DMEM) in the apical chamber and 600 μL in the basolateral chamber with 10% fetal bovine serum 1% (v/v), non-essential amino acids, 2 mM l-glutamine, 100 μg/mL streptomycin, and 100 U/mL penicillin. Media was changed every 24 h until cells reached confluence. Where indicated, Humolyte^®^ was added to the apical chambers to achieve a final concentration of 5 mg/mL of 2′-FL while control cells were treated with Gatorade (50% diluted in PBS and mixed in media) in the apical chambers for 48 h prior to the addition of 5 μg/mL of cisplatin (final concentration) diluted in DMEM and media to the basolateral chamber. The media was changed at 48 and 72 h, and assayed for different experiments so as measures of cell viability, mucin production, and cytokine secretion can be performed up to 96 h of treatment time.

#### 2.3.2 Cell viability assay

Was performed using CellTitre-Blue assay (Promega^®^) with conditions and cell culture set up with HT29-MTX cells under the above conditions (34). Total percentage of live cells were counted under these conditions; all experiments were performed in triplicate and repeated three independent times. Data represent mean viable cells (% normalized to control) averaged from three independent experiments.

#### 2.3.3 TEER assay

To determine the integrity of the confluent monolayer of Caco-2 cells, transepithelial electrical resistance (TEER) was measured using an epithelial volt-ohm-meter (EVOM^2^; WPI, Berlin, Germany) under various treatment conditions (35), including Humolyte^®^ and/or Gatorade in the apical chambers and cisplatin in the basolateral chambers.

#### 2.3.4 Mucin production

Was quantified through the Periodic Acid-Schiff’s (PAS) assay from the apical supernatants, using fresh media as the background control. PAS assay detects carbohydrates with adjacent hydroxyl groups and mucin is highly reactive. Mucin production with cisplatin treatment of goblet (HT29-MTX) cell line was quantified under each condition (Humolyte^®^ or Gatorade) at 0, 24, 48, 72, and 96 h from the apical supernatant in cells grown in transwell chambers and expressed as % of baseline control.

### 2.4 Statistics

*Animal studies*: Animals were weighed daily and weight (in g) expressed as mean with standard deviations were compared from day 6 (day of injection) to that on day 11 (euthanasia) between control and Humolyte^®^ treated groups for each of the four drugs using a two-way repeated measures analysis of variance (ANOVA) to account for multiple time points within treatment groups with *p* < 0.05 considered significant. Mean quantitative BM scores over the 10-day period are compared between control and Humolyte^®^ treatment groups for each chemotherapeutic agent for a particular day (time point) using the Mann–Whitney *U* (Wilcoxon rank-sum) test. Non-parametric data for total mean pathology scores were compared between control (water) and Humolyte^®^ (treatment) groups for each chemotherapeutic agent using the Mann–Whitney *U*-test with *p* < 0.05. Mean of goblet cells per high power field were compared between control and Humolyte^®^ treated group for each of the four drugs using one-way ANOVA with *p* < 0.05 considered significant.

For the *in vitro* studies depicting cell viability, barrier function, and measurement of PAS secretion, data are reported as mean ± standard deviation of three independent repeats. Two-way ANOVA with Tukey’s multiple analysis was performed to analyze statistical differences within the treatment groups at different time points as indicated.

**Figure 1:**
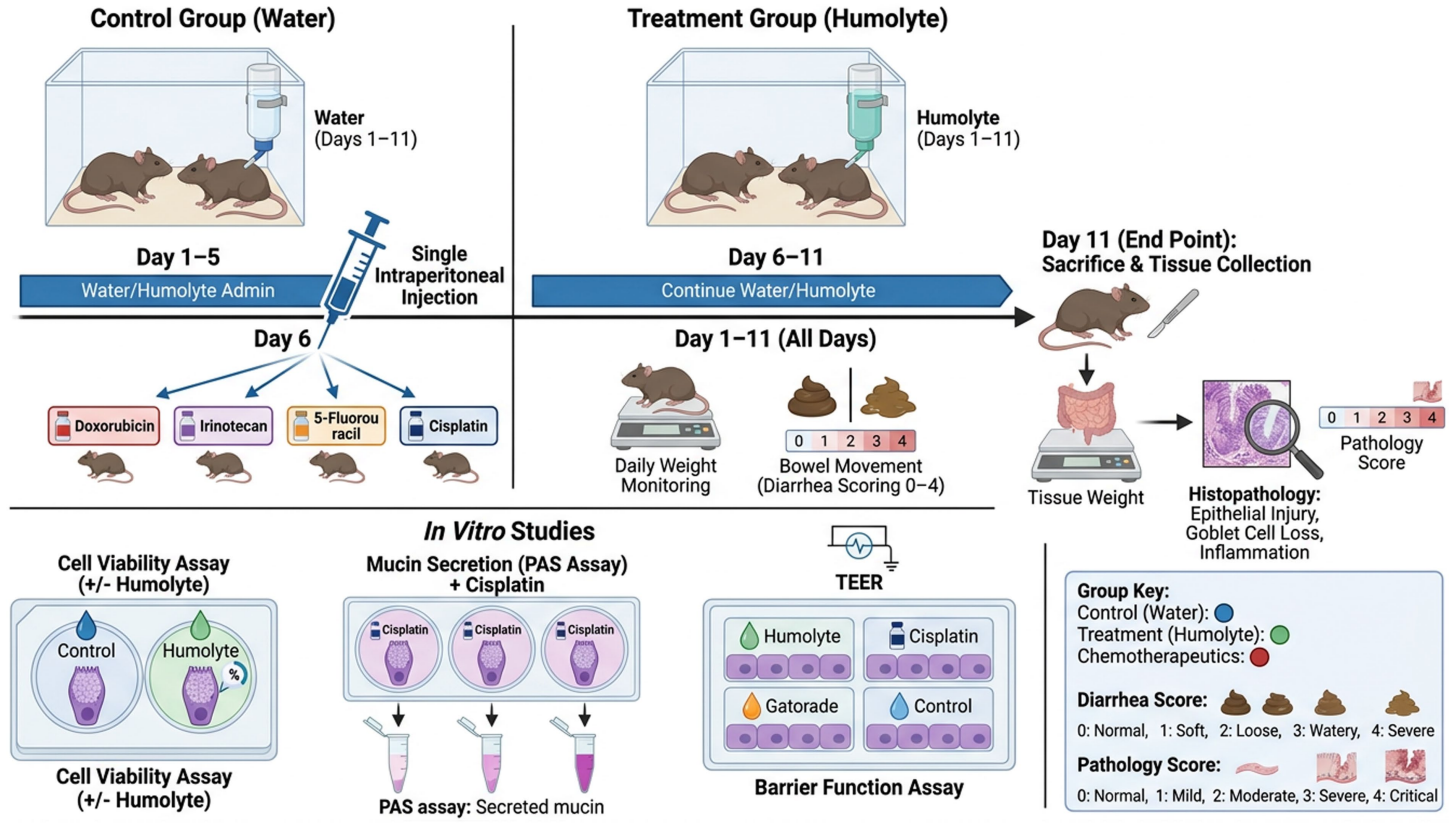
Overview of the experimental design of all the studies (animals and *in vitro*) as depicted in a graphical format. Figure is generated with the assistance of https://Figurelabs.ai. Details of experiments and design is described in methods.

Figure 1 graphically depicts the details of the experimental design for the studies conducted.

## 3 Results

### 3.1 Humolyte^®^ is well tolerated and reduces the severity of diarrhea in Wistar rats treated with chemotherapy

Studies indicate that Humolyte^®^ medical food is well tolerated in rats throughout the 10-day experimental period, beginning 5 days prior to chemotherapy. At baseline, all rats that were supplemented with Humolyte^®^ had normal bowel movements (scored 0) without diarrhea, and had similar growth in body weight compared with the control group supplemented with water (data not shown). This was not surprising, given that all ingredients in Humolyte^®^ (see Table 1) are certified as GRAS (generally recognized as safe) by the FDA and are used in humans, including infant nutrition.

Upon intraperitoneal injection of chemotherapeutic drugs, all animals developed diarrhea, except those treated with low-dose cisplatin. This dose was intentionally chosen to assess early and mild cellular changes (evident on histology) without inducing severe GI injury. All chemotherapeutic agents, particularly irinotecan and 5-FU, induced immediate and severe diarrhea within 24 h as depicted by a BM score of 3–4 in all animals receiving these two drugs (Table 2). However, the diarrheal score in these two groups was significantly lower (BM score of 0–1) in rats who were supplemented with Humolyte^®^ indicating a GI protective effect. Doxorubin therapy caused diarrhea after 36 h, which was also significantly lesser in the group that received pre-treatment with Humolyte^®^. Observational data indicated that animals that were supplemented with Humolyte^®^ consumed a normal amount of chow compared with the control animals receiving water during chemotherapy (data not shown).

**Table 2:** Bowel movement score of animals measured daily treated with chemotherapy drugs and those pre-medicated with Humolyte® medical food prior to the drug therapy.

Treatment	Mean bowel movement score (*n* = 6 in each group)									
	DAY 1	DAY 2	DAY 3	DAY 4	DAY 5	DAY 6	DAY 7	DAY 8	DAY 9	DAY 10
NC	0	0	0	0	0	0	0	0	0	0
HUM	0	0	0	0	0	0	0	0	0	0
DOX	0.00	0.00	0.00	0.00	0.00	0.00	0.67*	0.67*	0.83*	0.33*
HUM + DOX	0	0	0	0	0	0	0	0	0	0
IRI	0.00	0.00	0.00	0.00	0.00	0.00	3.00*	3.17*	3.50*	3.50*
HUM + IRI	0.00	0.00	0.00	0.00	0.00	0.00	2.33	2.83	2.33	1.67
5FU	0.00	0.00	0.00	0.00	0.00	1.83*	2.50*	2.50*	2.50*	2.50*
HUM + 5FU	0	0	0	0	0	0	0	0	0	0
CIS	0	0	0	0	0	0	0	0	0	0
HUM + CIS	0	0	0	0	0	0	0	0	0	0

At any given day, the mean BM score in rats treated with chemotherapeutic drugs was much lower in the Humolyte® group compared to group that received water for hydration.*Denotes *p* < 0.01 between the mean score on that particular day in the treatment (drug- DOX, IRI, 5FU or CIS with water for hydration) versus the same drug with Humolyte®. For example BM score on day 8 with IRI (3.17) is compared with HUM + IRI on day 8 (2.83).NC, normal control; HUM, Humolyte®; DOX, doxorubicin; IRI, irinotecan; 5FU, 5′-flurouracil; CIS, cisplatin.

### 3.2 Humolyte^®^ significantly mitigates the chemotherapy-induced weight loss in Wistar rats

**Figure 2:**
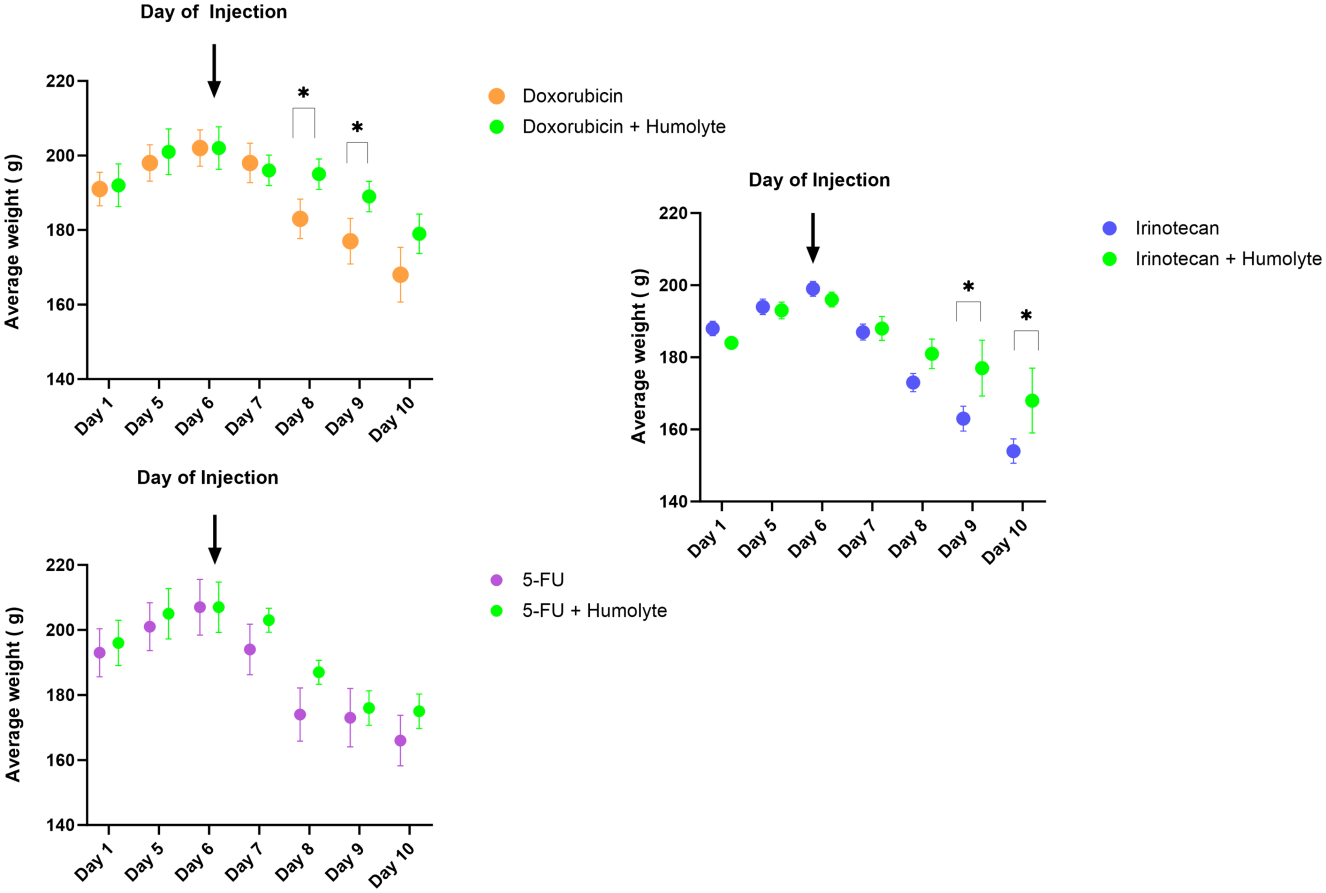
Changes in mean weight of Wistar rats treated with chemotherapeutic agents with (green) or without Humolyte^®^ supplementation. 8-week old rats (*n* = 6 per group) were either fed with normal chow and given water (control) ad lib or Humolyte^®^ for 5 days prior to the single intraperitoneal injection of chemotherapeutic drugs and weights were recorded every morning. The day (6) after the injection, all animals except in the cisplatin group progressively lost weight, most significantly with irinotecan, which also led to severe diarrhea. Humolyte^®^ supplementation significantly reduced drug induced weight loss. Expressed above are mean weights for each group on different days, ± SD; *p < 0.05. The lower dose for cisplatin was insufficient to induce significant changes in bowel function with no significant weight loss (data not shown).

Diarrhea can induce significant weight loss and cause malnutrition due to a deficiency of nutrients, electrolytes, and water. As expected, rats treated with chemotherapeutic drugs developed diarrhea that led to significant weight loss over the next 5 days (Figure 2). The irinotecan treated group was the most affected, with an average weight loss of approximately 44.6 g (SD ± 6.57) from the day of injection. However, the mean weight loss was markedly lesser at 25.2 g (SD ± 12.1) in irinotecan group that was supplemented with Humolyte^®^. In the 5-FU and doxorubicin treatment groups, a similar trend was observed with the control group having a mean weight loss of 41.1 g (SD ± 18.7) with 5-FU which was much lesser at 35.7 g (SD ± 13.3) with Humolyte^®^ treatment, whereas the doxorubicin treated animals had a mean weight loss of 32.7 g (SD ± 18.6) over 5 days without supplementation which was improved to 26 g (SD ± 13.5) with Humolyte^®^ pretreatment. This indicated that Humolyte^®^ reduced the extent of weight loss in Wistar rats, likely by reducing diarrhea and maintaining nutrition as well as through an improvement of overall gastrointestinal function. As expected, since cisplatin did not cause diarrhea at 5 mg/kg dose, these animals did not lose any significant weight. At day 11, two animals died in irinotecan group and one in 5-FU group supplemented with water, whereas all animals supplemented with Humolyte^®^ were alive regardless of the chemotherapeutic drug used.

### 3.3 Humolyte^®^ significantly reduces the extent of GI injury from mucositis in rats treated with chemotherapy

**Figure 3:**
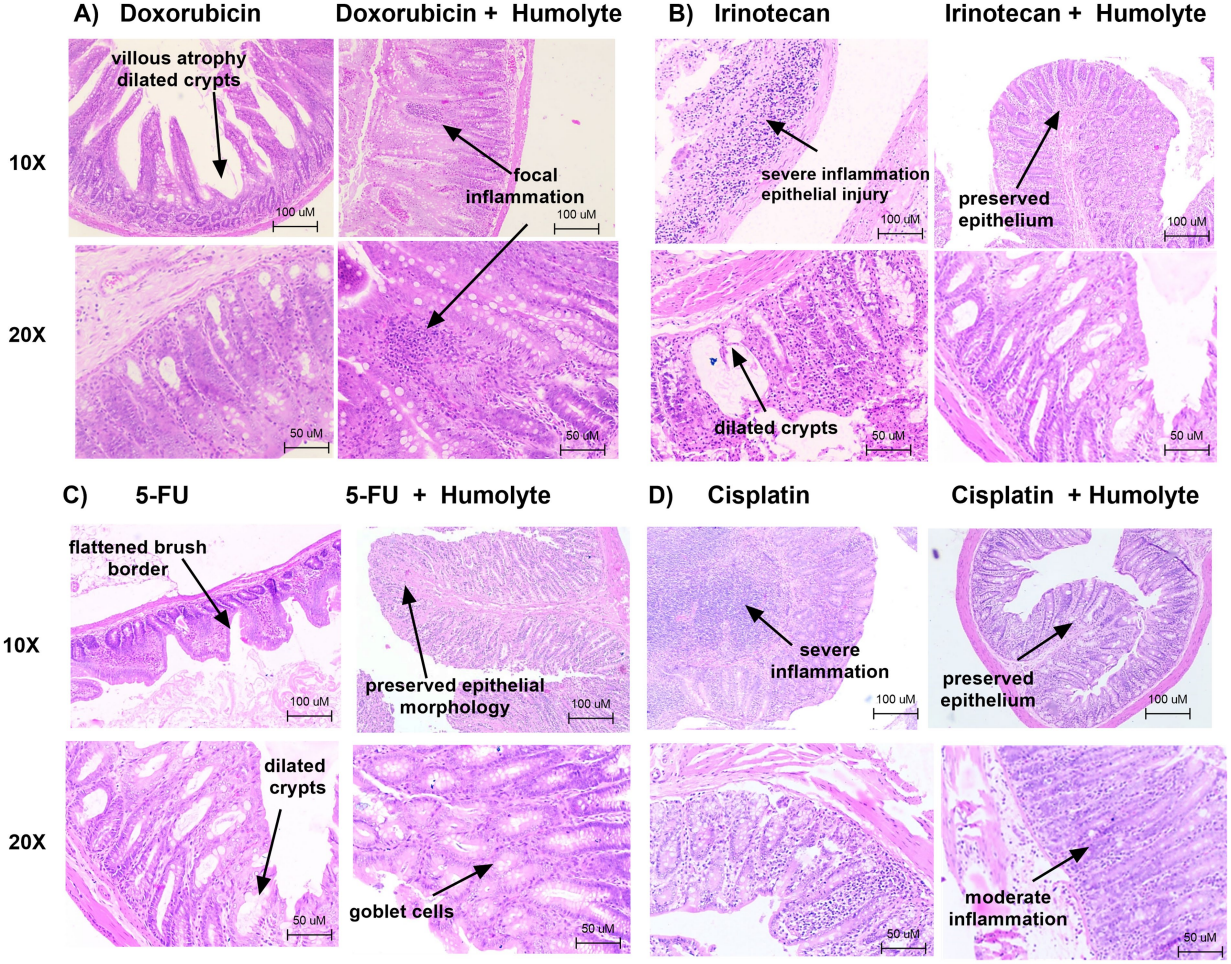
Humolyte^®^ supplementation reduces gastrointestinal mucosal injury from chemotherapeutic agents in Wistar rats. Representative images of ileo-colonic mucosa from each of the animals after treatment with different chemotherapeutic drugs (*n* = 6 per group) with and without Humolyte^®^ supplementation. The group treated with doxorubicin **(A)** and 5-FU **(C)** had significant crypt dilation, loss of goblet cells, flattened brush border and loss of villi, while irinotecan injection **(B)** led to significant mucosal injury depicted by loss of villar architecture and severe inflammation all of which was mitigated with prior supplementation with Humolyte^®^. Cisplatin caused significant inflammation **(D)** which was markedly reduced in presence of Humolyte^®^. Shown above are hematoxylin/eosin-stained representative images sampled from at least 3 distinct areas per slide each at a 10X and 20X magnification with scale bars as indicated.

**Figure 4:**
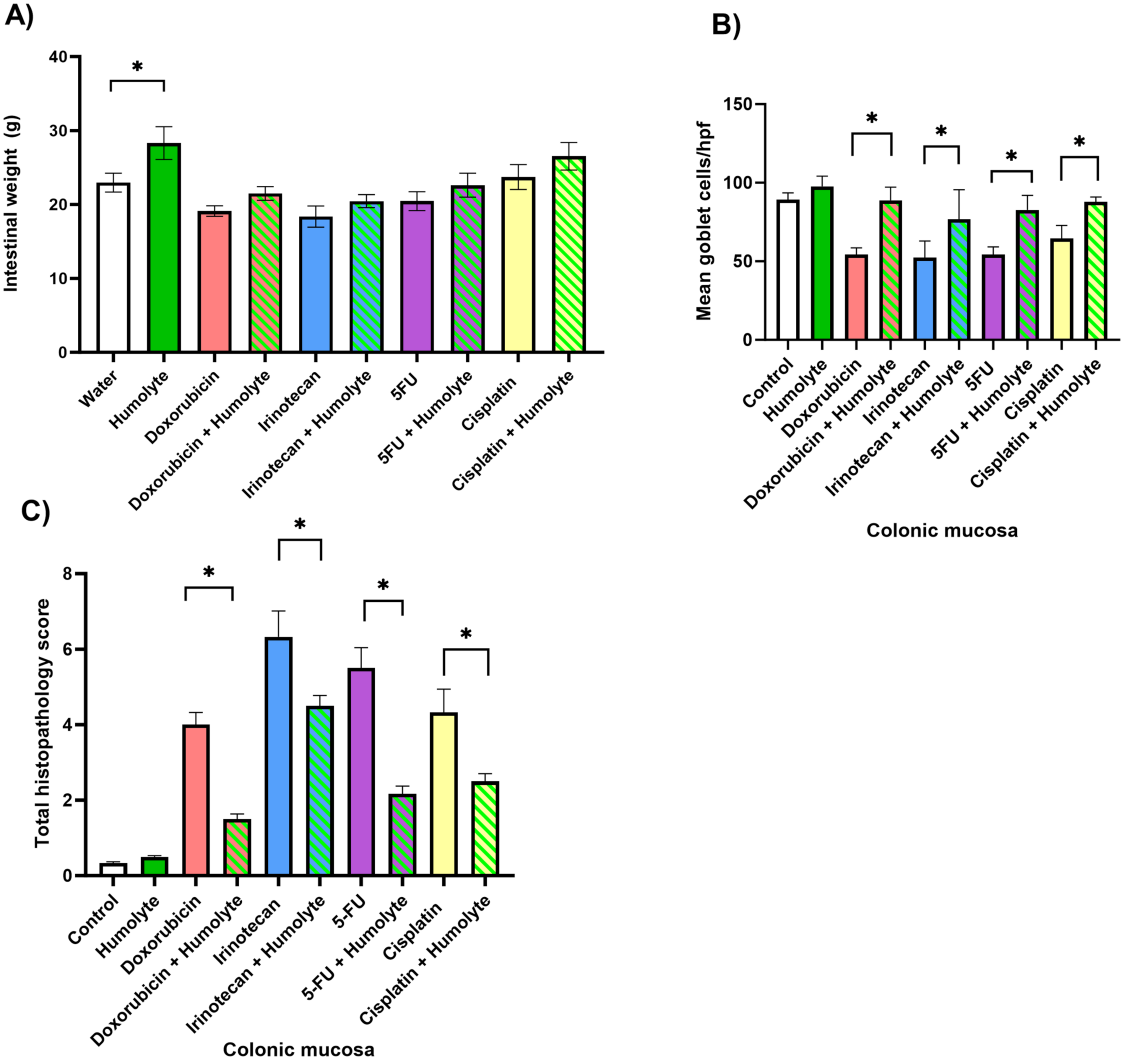
Total intestinal weight and quantitative cumulative pathological scores for ileo-colonic mucosal damage with chemotherapeutic drugs with and without Humolyte^®^ supplementation. Animals (*n* = 6 per treatment arm) that received Humolyte^®^ beginning 5 days prior to injection with chemotherapy had a higher total intestinal weight (normalized to today body weight of the animal) depicted in **(A)**, a higher mean goblet cell count/hpf as shown in **(B)**, and a lower total pathological scores shown in **(C)** indicating a lesser extent of tissue injury, inflammation and a more preserved GI mucosal architecture than those who received water (control). Three distinct areas per colon were sectioned from each animal within the treatment groups and three distinct areas per slide were counted and averaged. Cumulative pathology scores are expressed from 0 (no damage) to 4 (severe disruption) based on pathological findings as described in Methods. Expressed above is the mean total cumulative score obtained from summation of all pathological individual scores for different parameters represented with standard deviation for ileo-colonic tissue in each treatment group with and without Humolyte^®^; *p < 0.05.

To assess the extent of epithelial injury and resulting inflammation, animals were euthanised humanely on day 11 and histopathology of the oral and GI tissue was examined by a pathologist after hematoxylin and eosin (H/E) staining at 10× and 20× magnification. As depicted, all chemotherapeutic drugs induced significant GI damage but through different mechanisms collectively characterized by epithelial cell injury and necrosis, loss of tubular brush border, flattening of intestinal villi, reduction in the depth of crypts, loss of goblet cells, interstitial edema, and, in some cases, vascular thrombosis (Figure 3). A significant influx of inflammatory cells in the lamina propria was observed, particularly in the irinotecan group. Both, the inflammation and the extent of injury, were markedly reduced in animals pre-treated with Humolyte^®^. In particular, Humolyte^®^ supplementation maintained the villar architecture, preserved goblet cells, and reduced the inflammatory infiltrates in animals treated with all four chemotherapeutic agents (Figures 3A–D). In cisplatin treated group, significant patchy inflammation was evident despite a lack of frank epithelial injury; the extent of this inflammation was reduced in Humolyte^®^ supplemented group (Figure 3D). Additionally, the intestinal weight of animals pre-treated with Humolyte^®^ was higher than those in the control group possibly from the improvement in colonic mass (either from microbiome and/or mucin production), although this did not reach statistical significance (Figure 4A). Quantitative assessment of goblet cell counts in colonic tissue of animals indicated a significantly higher number per hpf in Humolyte^®^ supplemented group (Figure 4B). Similarly, ileo-colonic histopathology demonstrated a markedly lower tissue injury score in animals supplemented with Humolyte^®^ indicating a cytoprotective effect of 2’-FL in presence of chemotherapy (Figure 4C).

**Figure 5:**
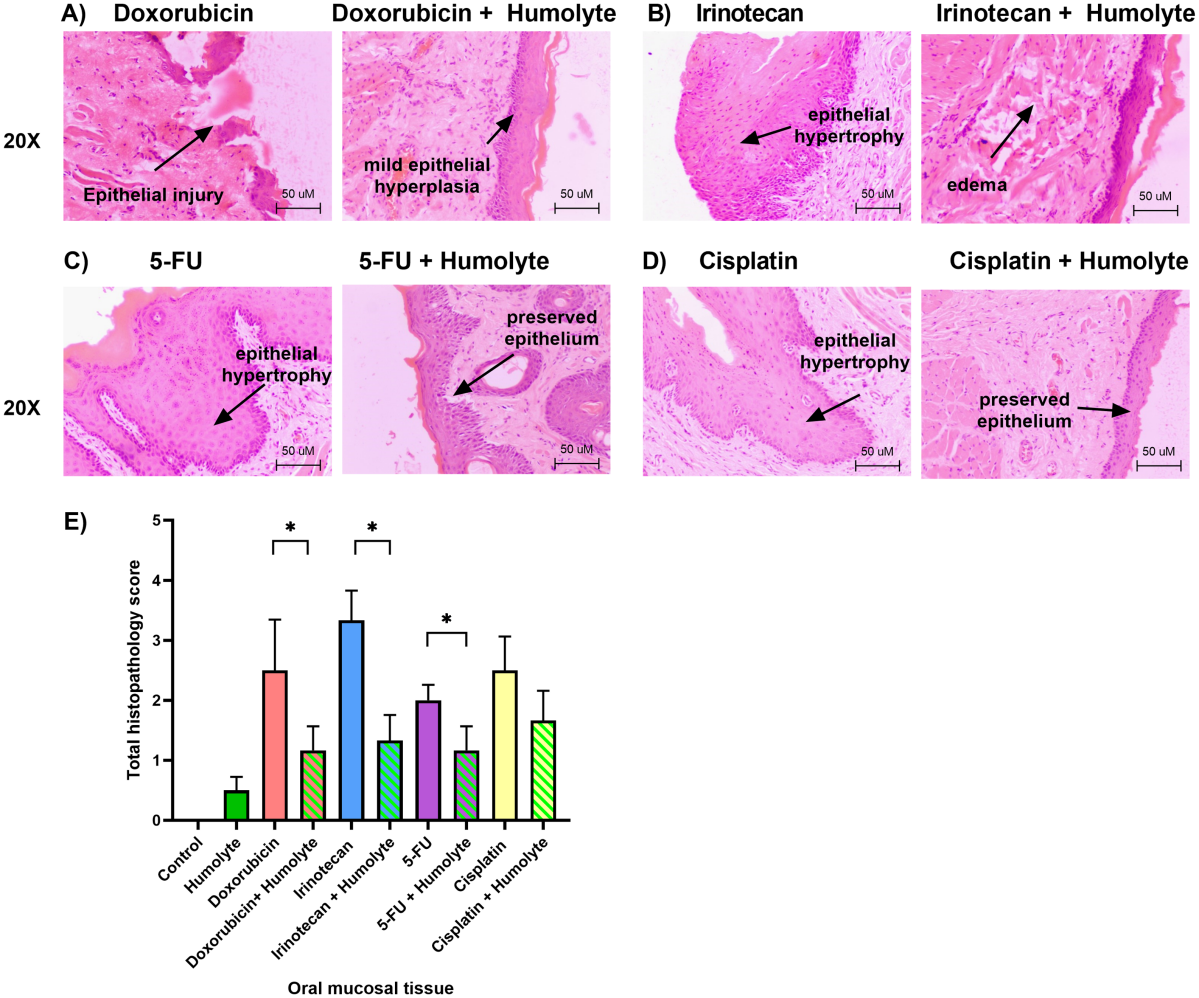
Humolyte^®^ supplementation reduces the severity of oral mucosal injury from chemotherapeutic agents. In the oral mucosa, all chemotherapeutic drugs caused marked epithelial hypertrophy and mucosal injury (particularly with doxorubicin) which was significantly lower in animals supplemented with Humolyte^®^ **(A–D)**. Represented above are images from oral tissue from animals (*n* = 6) treated by four distinct chemotherapeutic drugs with or without Humolyte^®^ pre-hydration. **(E)** demonstrates the mean total cumulative score obtained from summation of all pathological individual scores for different parameters represented with standard deviation for oral tissue in each treatment group with and without Humolyte^®^; *p < 0.05.

These protective effects were also evident in oral mucosal tissue (Figures 5A–D). Humolyte^®^ preserved the epithelial barrier, reduced the extent of oral epithelial hyperplasia, and minimized mucosal ulceration in response to all four drugs, particularly doxorubicin, which was also reflected in a favorable quantitative histopathology score (Figure 5E).

### 3.4 Humolyte^®^ improves cell viability *in vitro* and reduces epithelial monolayer permeability through improvement in barrier function

**Figure 6:**
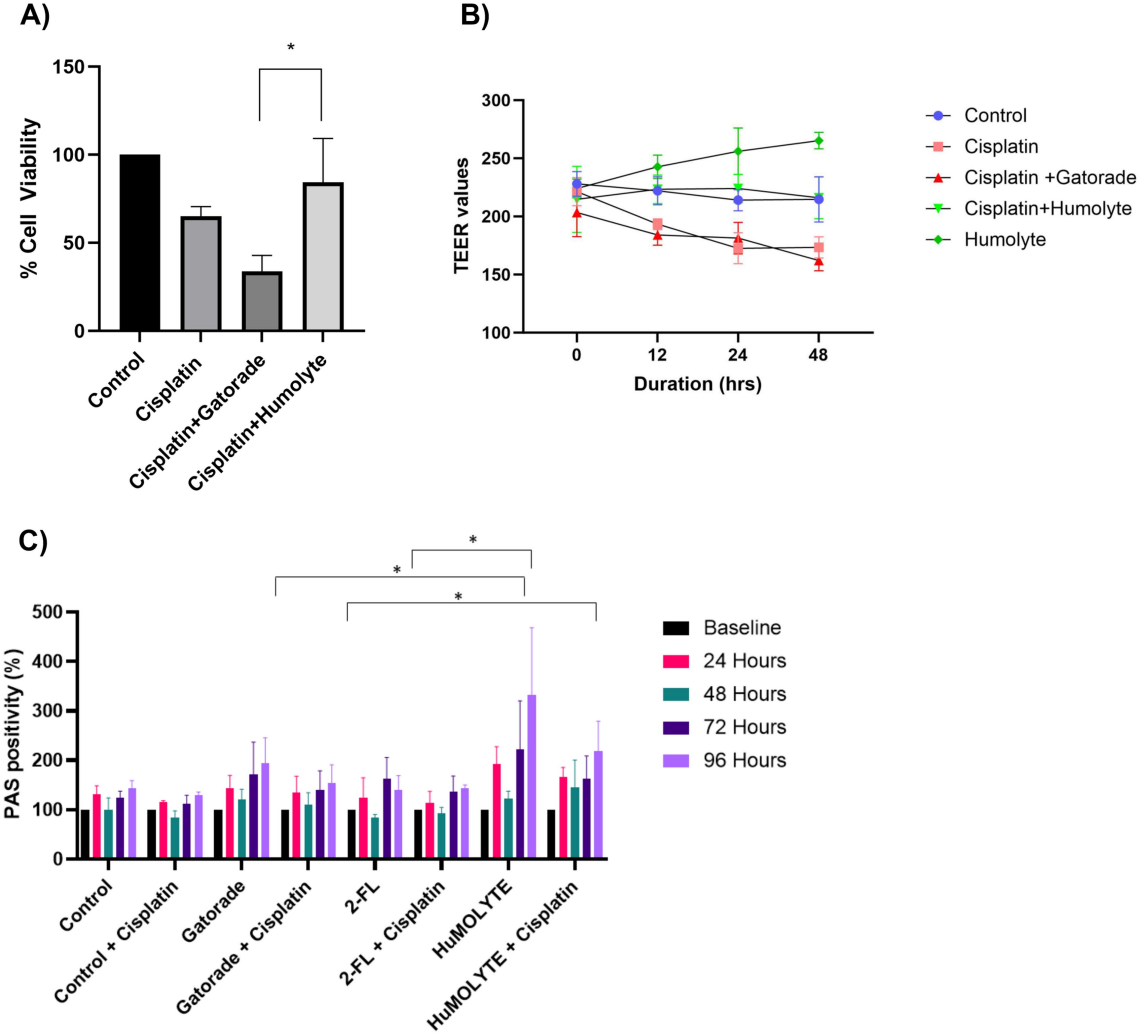
Humolyte^®^ improves intestinal cell viability, barrier function and mucin production in vitro. **(A)** HT29-MTX goblet cells grown in transwell chambers treated with Humolyte^®^ for 48 h show improved survival than Gatorade^®^ when exposed to cisplatin 5 μg/mL in the basolateral chambers when assessed using CellTiter assay. Expressed is % of viable cells from baseline in each group, compared with ANOVA, *p < 0.05. **(B)** Caco-2 monolayer subjected to TEER assay demonstrates improvement in monolayer integrity with Humolyte^®^, both at baseline and in the presence of cisplatin. Gatorade^®^ does not improve epithelial monolayer barrier function as reflected with markedly lower TEER values indicating increase in permeability. Values indicate means in each group with SD. **(C)** Quantitative measure of mucin production using PAS assay from HT29-MTX goblet cells in transwell culture in the presence of cisplatin and Humolyte^®^. At 96 h, cells treated with Humolyte^®^ significantly upregulate mucin production more than treatment with 2′-FL alone. Data are % normalized to baseline and analysed with ANOVA, *p < 0.05.

To assess the mechanisms by which the 2’-FL electrolyte mix can improve GI injury, we cultured H29-MTX goblet cells for 21 days until they formed a monolayer and assessed their viability in presence of the chemotherapeutic drug cisplatin. Where indicated, cells were pre-treated with Humolyte^®^ or Gatorade^®^ (which has electrolytes only) in the culture media, and cell viability was assessed. As shown in Figure 6A, cisplatin significantly reduced cell survival, an effect worsened in cells treated by Gatorade^®^. However, pre-treatment with Humolyte^®^ significantly reduced cisplatin toxicity, improving goblet cell viability in culture.

Next, this study tested whether 2′-FL in Humolyte^®^ increased cell–cell adhesion in the presence of cisplatin using a TEER (transepithelial electrical resistance) assay, which measures intestinal permeability. As expected, cisplatin significantly increased the intestinal permeability, which was improved back to baseline in the presence of Humolyte^®^ (Figure 6B). However, electrolyte supplement such as Gatorade^®^, which lacks 2′-FL, did not improve the permeability of the adherent epithelial monolayer. As expected, the monolayer of cells treated with Humolyte^®^ alone, without cisplatin, showed the least permeability and greatest adherence, indicating improved barrier function.

### 3.5 Humolyte^®^ increases production of mucin by goblet cells *in vitro*

H29-MTX cells were cultured for 21 days in presence of cisplatin and supplemented with either Gatorade^®^ or Humolyte^®^ were assessed for mucin production in the supernatant using the PAS assay. The mucin production is increased over a 96-h period in all groups, but to a lesser extent with cisplatin treatment (Figure 6C). Humolyte^®^ markedly increased the mucin production at baseline and also in all the cisplatin treated groups. Interestingly, this stimulation exceeded that of 2′-FL treatment alone, indicating a synergistic effect of electrolytes (particularly magnesium) on mucin production *in vitro*. These findings indicate that the 2′-FL-based medical food, Humolyte^®^, improves epithelial cell viability and barrier function, enhances goblet cell survival, and increases mucin secretion.

## 4 Discussion

This study highlights the potential novel application of the ingredient, 2’-FL, in promoting the gut health of patients exposed to chemotherapy. Presently, there are no specific preventive measures or agents that consistently reduce the incidence and severity of oral and intestinal mucositis during chemotherapy. Palifermin, a keratinocyte growth factor, is not widely used for mucositis prevention due to lack of significant efficacy studies, high cost, and safety concerns (36). L-glutamine has been shown to reduce the severity of mucositis in patients with head and neck cancer, but its efficacy in other solid tumors or hematological malignancies is questionable. Additionally, L-glutamine is not used as a hydration solution and its absorption from the GI tract can complicate its effectiveness (37). Thus, mucositis is commonly managed by local oral care, hydration, and supportive therapies (such as ice chips, saline rinses, pain killers, and home remedies such as honey). These are not significantly and consistently effective in preventing oral and intestinal lesions, and the toxicity of chemotherapeutic drugs towards the GI tract and the microbiome goes unchecked. In addition to early debilitating effects of GI toxicity from barrier dysfunction, some long-term consequences of chemotherapy may be mediated through its deleterious impact on the microbiome.

Studies indicate that chemotherapeutic agents cause significant GI cell injury, which can be significantly mitigated by oral hydration mix enriched with magnesium and 2′-FL. The efficacy of this formulation was observed against the off-target GI effects of several commonly used chemotherapeutic drugs. This hydration mix containing magnesium and 2′-FL, improved epithelial cell survival and mucin secretion from goblet cells, translating into reduced GI injury and inflammation *in vivo*. This is not surprising, since magnesium is anti-apoptotic, is vital in cell survival and is highly anti-inflammatory at a cellular level (38). Since chemotherapeutic drugs cause significant GI and renal magnesium wasting (38), it is possible that subsequent rounds of chemotherapy can be more deleterious to a magnesium depleted protoplasm leading to quicker rates of apoptosis and GI cell injury. Interestingly, 2′-FL strengthens cell–cell adhesion and stimulate mucin secretion (39), indicating a potentially beneficial effect of combination therapy for patients who lose magnesium due to chemotherapy-induced GI tract injury. A stronger GI luminal surface can improve the absorption and retention of magnesium, electrolytes, and water and can improve dehydration. Improved oral intake of water with electrolytes enhances epithelial cell survival through luminal hydration, allowing improvement in 2′-FL transit through the GI tract and its delivery into the colon where it is metabolized by the microbiota. A robust microbiome can produce short chain fatty acids which in turn augment the intestinal barrier function by increasing mucin secretion thereby strengthening the feedback loop. Future studies should aim at comparing the differences in GI injury in rats supplemented with 2′-FL prebiotic alone in comparison with Humolyte^®^ to delineate the effects of the HMO versus electrolytes alone. Due to the short duration of therapy, we did not evaluate the effects of Humolyte^®^ on the microbiome under these conditions, but will be the focus of a longer study in the future.

This study showed that rats supplemented with Humolyte^®^ had significantly milder diarrhea across all conditions, particularly with diarrheagenic drugs such as irinotecan and 5-flurouracil. These animals wasted less water from GI tract, maintained a stable appetite, and preserved higher weight compared with the control animals receiving only water during chemotherapy. At baseline, despite a higher magnesium content of the medical food, none of the animals exhibited diarrhea indicating good tolerance to the supplement. Visual changes of oral mucositis or ulcers were not clearly observed in rats with chemotherapy despite the histopathological alterations on microscopy, likely due to species specific differences of oral/GI mucosal tissue and microbiome composition.

Tissue pathology indicated lower inflammatory scores and lesser tissue damage in rats supplemented with Humolyte^®^ in all treatment conditions. The inflammatory infiltration was reduced in cisplatin treated group despite the fact that tissue injury at that dose was not severe. This dose was intentionally lower to assess the tissue specific changes with lower doses of chemotherapy. The 2′-FL in Humolyte^®^ significantly increased intestinal weight compared with water alone, suggesting an increase in the colonic mass (likely from mucin, given the short duration) confirming previously reported findings by other studies (39). Future studies should aim at characterizing the cytokine changes at the tissue level at different time points (days 1–5 after chemotherapeutic injections) to delineate the mechanisms of injury. Additionally, temporal changes in MUC2 gene expression under chemotherapy may be assessed to further define the mechanisms of 2′-FL mediated mucin production (40).

The *in vitro* mechanistic studies indicated that Humolyte^®^ preserved the epithelial cell adhesion, and improved goblet cell survival and function (such as mucin secretion) under stress conditions, such as exposure to a high-glucose hydration mix, Gatorade^®^. Measurements of different cytokines such as TNF-alpha, IL-6, and IL-10 under these conditions may be critical to define mechanisms and will be undertaken with longer duration of *in vitro* assays, including exposure to multiple chemotherapeutic agents. It is possible that such changes are transient and temporally spaced after chemotherapy.

Although the initial findings in this pilot experiments are encouraging, several limitations should be noted. First, the obvious limitation from animal models, particularly a single species with a distinct diet, microbiome, and the GI architecture cannot directly be applied to humans without validation through robust clinical trials. Second, majority of cancer patients receive a combination of chemotherapeutic drugs. The effect of this formulation may be blunted in the presence of multiple cytotoxic drugs and may require a dose adjustment compared with that used in this study. Third, to address this concern, a clinical trial is presently underway using this medical food that will include pretreatment of patients with solid tumors who are deemed at a higher risk of developing mucositis from the combination chemotherapy. Finally, the study may also include serial measurements of serum electrolytes at different time points, particularly magnesium, to demonstrate whether 2′-FL in Humolyte^®^ enhances absorption of these minerals from the GI tract that are eventually reflected by improved serum levels.

## 5 Conclusion

This study indicates that an oral rehydration mix with 2′-fucosyllactose HMO can reduce the extent of GI and oral mucositis across a variety of chemotherapeutic agents. Electrolytes, particularly magnesium in the mix, exert a beneficial effect on intestinal cell survival and function both *in vitro* and *in vivo.* Importantly, 2′-FL may be safely delivered in a flavored hydration mix, thereby reducing the pill burden for patients who often have difficulty in swallowing during mucositis episodes. These pre-clinical studies have profound implications for the development of a safe, food-grade, and economical solution for the oral hydration and electrolyte repletion during chemotherapy. The formulation may reduce the burden of mucositis, a debilitating side effect of chemotherapy with limited therapeutic options.

## Data Availability

The raw data supporting the conclusions of this article will be made available by the authors, without undue reservation.
